# Fibrinogen and Fibrin as Growth Factor Regulators: Pathological Implications, and Translational Opportunities

**DOI:** 10.3390/biom16020335

**Published:** 2026-02-23

**Authors:** Abha Sahni, Sanjeev K. Sahni

**Affiliations:** Department of Pathology, University of Texas Medical Branch at Galveston, 301 University Blvd, Galveston, TX 77555, USA; sksahni@utmb.edu

**Keywords:** fibrinogen, growth factors, thrombosis, therapeutics, cancer

## Abstract

Fibrinogen and fibrin are multifunctional plasma proteins that play central roles in hemostasis, tissue repair, and extracellular matrix organization. Their complex molecular architecture enables specific interactions with key growth factors, including fibroblast growth factor-2 (FGF-2), vascular endothelial growth factor (VEGF), platelet-derived growth factor (PDGF), transforming growth factor-β (TGF-β), and others, promoting growth factor localization, protection from proteolysis, and enhanced signaling. These interactions regulate essential biological processes such as angiogenesis, cell proliferation, and wound healing. Dysregulation of fibrinogen–fibrin contributes to pathological conditions, including thrombosis, chronic inflammation, cancer progression, neurological complications, and impaired tissue regeneration. Recent advances in fibrin-based biomaterials leverage these molecular interactions for controlled therapeutic delivery and regenerative medicine applications. Emerging recombinant fibrinogen technologies and precision biomaterial engineering further expand the translational potential of targeting fibrinogen–fibrin growth factor interactions to improve clinical outcomes. This review offers an integrated overview of fibrinogen and fibrin biology, detailing their molecular interactions with growth factors, their pathological implications, clinical significance, and future research directions, emphasizing the translational potential of leveraging these interactions to advance human health.

## 1. Introduction

Fibrinogen and its polymerized form, fibrin, are essential plasma proteins traditionally recognized for their roles in hemostasis and clot formation but increasingly appreciated as active regulators of tissue repair and regeneration. Fibrinogen is a soluble hexameric glycoprotein composed of Aα, Bβ, and γ chains arranged into a symmetric molecule with a central E domain and two distal D domains [1,2]. Upon vascular injury, thrombin cleaves fibrinopeptides A and B, triggering fibrin polymerization into a three-dimensional, factor XIIIa–stabilized network that serves as a provisional extracellular matrix (ECM) [3]. Beyond providing structural support for clot formation, fibrin(ogen) functions as a dynamic signaling platform through its capacity to bind and modulate many growth factors. Fibrinogen and fibrin interact with key regulators of tissue repair, including fibroblast growth factor-2 (FGF-2) [4,5], vascular endothelial growth factor (VEGF) [5,6], and platelet-derived growth factor (PDGF) [5], placenta growth factor (PlGF) [5], Transforming growth factor-β (TGF-β) [5] and Interleukin 1 (IL-1β) [7]. These interactions enable localized sequestration of growth factors within fibrin matrices, protecting them from proteolytic degradation and establishing spatial gradients that enhance receptor-mediated signaling. Importantly, fibrin polymerization exposes cryptic binding sites that increase growth factor affinity, thereby coupling coagulation to growth factor activation at sites of injury. The interaction of key growth factors with fibrinogen and fibrin is summarized in Table 1 below.

Through these mechanisms, fibrin-bound growth factors coordinate essential biological processes, including angiogenesis, endothelial and fibroblast proliferation, and wound healing. Tight regulation of fibrinogen–growth factor interactions is critical, as their dysregulation contributes to pathological outcomes including chronic inflammation, fibrosis, tumor angiogenesis, and impaired tissue repair. Fibrin serves as both a provisional extracellular matrix and a regulator of inflammatory signaling following tissue injury. During acute wound healing, fibrin deposition localizes cytokines and growth factors, including VEGF, FGF-2, PDGF, PlGF, and TGF-β, while providing adhesive cues for immune and endothelial cells. This matrix-mediated sequestration restricts diffusion, protects labile mediators from rapid degradation, and enables their controlled, proteolysis-dependent release as fibrinolysis and tissue remodeling proceed, thereby coordinating inflammatory cell recruitment, vascular permeability, angiogenesis, and the transition toward repair. In contrast, chronic inflammatory conditions are characterized by persistent fibrin deposition resulting from ongoing vascular leakage or impaired fibrinolysis, leading to sustained integrin-mediated cell adhesion and prolonged growth factor retention within the matrix. Under these conditions, dysregulated release kinetics and continued exposure to fibrin-bound growth factors promote aberrant vascular activation, fibroblast accumulation, and excessive extracellular matrix production, while impairing inflammatory resolution. Thus, failure to appropriately remodel fibrin matrices converts a transient inflammatory scaffold into a persistent signaling platform that reinforces chronic inflammation and tissue pathology. Leveraging these molecular interactions, fibrin-based biomaterials have emerged as powerful platforms for controlled growth factor delivery in regenerative medicine, enabling precise temporal and spatial control of signaling. This review focuses on the molecular basis of fibrinogen and fibrin interactions with growth factors, their biological consequences in health and diseases, and emerging therapeutic strategies that exploit these interactions for tissue regeneration and targeted clinical interventions.

## 2. Fibrinogen and Fibrin: Synthesis, Structure, and Post-Translational Modifications

Fibrinogen is a multifunctional plasma glycoprotein essential for hemostasis, wound repair, and tissue remodeling. Synthesized predominantly in the liver, it circulates at high concentrations (2–4 g/L) in human plasma and is composed of three pairs of polypeptide chains—Aα, Bβ, and γ—linked by disulfide bonds to form a 340 kDa hexameric structure [10]. The molecule features a central E domain flanked by two D domains and coiled-coil connectors, which provide both structural stability and sites for molecular interactions critical during clot formation and platelet aggregation [11].

Fibrinogen synthesis is tightly regulated at transcriptional and translational levels and responds dynamically to constitutive and inflammatory signals [1,12]. Alternative splicing generates variants such as the γ′ chain, which constitutes roughly 8% of the γ chain population and confers specialized functional properties, including enhanced binding to plasma factor XIII [13]. The molecular architecture of fibrinogen—with 610 residues in the Aα chain, 461 in Bβ, and 411 in the γA chain—features disulfide bridges that maintain twofold symmetry and allow the molecule to elongate into 45 nm structures, supporting site-specific interactions with platelets, integrins, and other components of the coagulation cascade [1,14]. Clot formation begins when thrombin cleaves fibrinopeptide A from the Aα chains, exposing EA polymerization sites, followed by cleavage of fibrinopeptide B, which exposes EB sites. These sites interact with complementary regions in adjacent fibrinogen molecules (Da and Db in the D domain), forming double-stranded fibrils and branched junctions. Lateral associations, mediated in part by αC domains, generate ok.thicker fibers and a robust three-dimensional network [15]. Factor XIIIa enzymatically crosslinks fibrin via ε-(γ-glutamyl)lysine bonds, producing γ chain dimers, trimers, and higher-order structures that reinforce mechanical stability [16]. The microarchitecture of the resulting fibrin matrix, including fiber thickness, branching, and porosity, is influenced by thrombin concentration, with slower cleavage favoring more branched, elastic networks [17,18].

Post-translational modifications (PTMs) play an important role in shaping the structure and biological functions of fibrinogen and fibrin, thereby influencing their interactions with growth factors [19,20,21,22]. Modifications such as phosphorylation, oxidation, nitration, glycation, and enzymatic crosslinking can alter fibrinogen conformation, charge distribution, and susceptibility to proteolysis, with direct consequences for fibrin polymerization and matrix architecture [23]. Changes in network density or fiber organization are likely to affect the accessibility and affinity of growth factor–binding sites, influencing growth factor sequestration, protection from degradation, and release kinetics [24,25,26,27]. In pathological settings, such as inflammation, diabetes, or oxidative stress, disease-associated PTMs further remodel fibrin networks, often in a context-dependent manner that is difficult to generalize. Although systematic analyses of PTM-specific effects on growth factor binding remain limited, available evidence suggests that PTMs represent an additional regulatory layer linking biochemical environment to growth factor availability and tissue responses [20].

These structural features directly underpin fibrinogen and fibrin’s diverse biological functions, from hemostasis and wound healing to modulation of inflammation and tissue repair. Variations in fibrinogen concentration or structure, including genetic variants like γ′ fibrinogen, can predispose individuals to bleeding disorders, thrombosis, or impaired tissue regeneration [28]. Understanding these molecular mechanisms provides critical insight for therapeutic interventions, including fibrin-based biomaterials and targeted modulation of coagulation and tissue repair pathways.

## 3. Growth Factors in Tissue Repair

Growth factors are critical signaling proteins that regulate tissue repair and angiogenesis by binding to specific receptors on target cells, thereby activating intracellular pathways controlling migration, proliferation, differentiation, and ECM synthesis [29,30]. Their coordinated action is essential for restoring tissue integrity after injury. Key growth factors in wound healing include vascular endothelial growth factor (VEGF), fibroblast growth factor-2 (FGF-2), platelet-derived growth factor (PDGF), transforming growth factor-beta (TGF-β), and epidermal growth factor (EGF) [9]. VEGF primarily drives angiogenesis, stimulating endothelial cell proliferation, migration, and survival, particularly under hypoxic or inflammatory conditions [31]. FGF-2 promotes fibroblast and endothelial cell proliferation, ECM deposition, and matrix remodeling through induction of metalloproteinases [32,33]. PDGF, released rapidly by platelets, recruits fibroblasts and smooth muscle cells, aiding granulation tissue formation and stabilizing nascent vessels via pericyte recruitment [34]. TGF-β regulates inflammation, matrix deposition, and scar formation, balancing fibrosis with regeneration [35,36], while EGF accelerates re-epithelialization by promoting epithelial cell proliferation and migration [37].

These growth factors function in a tightly coordinated, phase-specific manner throughout wound healing—from hemostasis and inflammation to proliferation and remodeling. VEGF and FGF-2 drive vascular growth in the proliferative phase, whereas PDGF stabilizes the newly formed vascular network. Dysregulation of growth factor signaling contributes to chronic wounds, excessive scarring, or impaired healing seen in conditions such as diabetes or vascular disease. Therapeutically, exogenous administration or controlled delivery of growth factors—alone or in combination—has been used to enhance repair, with recent strategies leveraging biomaterials like hydrogels and scaffolds to mimic natural release kinetics. Multi-factorial approaches combining VEGF, FGF-2, PDGF, and EGF more closely replicate physiological repair processes and often yield superior regenerative outcomes.

## 4. Molecular Interaction Between Growth Factors and Fibrin(ogen)

**Figure 1:**
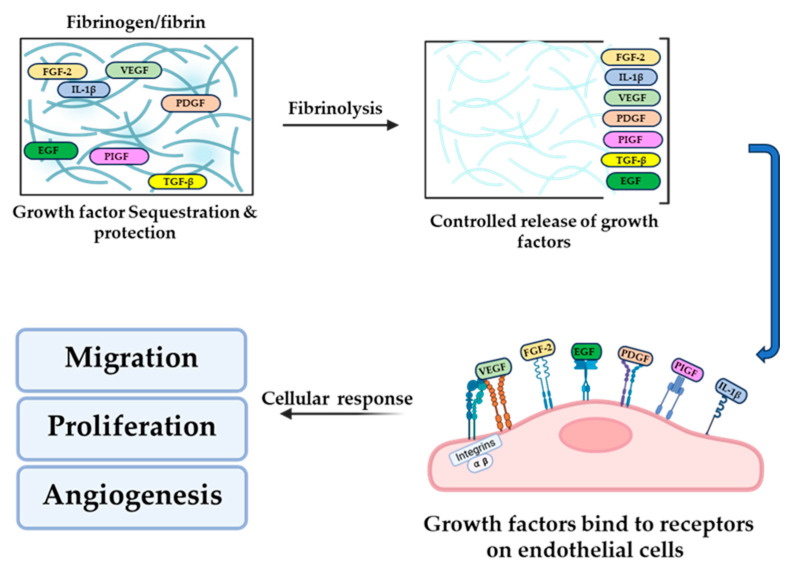
Fibrinogen/Fibrin-Mediated Storage, Release, and Bioavailability of Growth Factors. A schematic illustration shows how fibrin(ogen) controls growth factor availability and cellular responses during wound repair. Soluble fibrinogen and the insoluble fibrin network form a provisional matrix that sequesters multiple growth factors, including VEGF, FGF, PDGF, and TGF-β, along its fibers, concentrating them locally while protecting them from rapid degradation. Progressive fibrinolysis remodels the matrix and enables controlled release of bound growth factors, which then engage growth factor receptors on adjacent endothelial cells. Concurrently, integrin-mediated interactions at the fibrin–cell interface support adhesion and signaling. Together, matrix degradation and receptor activation drive downstream biological outcomes, including endothelial cell migration, proliferation, and angiogenesis. The illustrations are created using BioRender software (Sahni A., https://app.biorender.com/illustrations/69431ba20505fa63df077a7b (accessed on 19 February 2026)).

Fibrinogen and fibrin play a central role in coordinating hemostasis, ECM dynamics, and tissue regeneration by interacting with growth factors. These interactions localize, protect, and enhance growth factor signaling, directly influencing angiogenesis, cell proliferation, and wound healing. Fibrin(ogen) contains high-affinity binding domains for several growth factor families, including FGF, VEGF, and PDGF. Notably, the heparin-binding domain on the β-chain of fibrinogen (amino acids 15–66) interacts with positively charged regions on growth factors, with lysine and arginine residues being critical for binding [5]. For instance, FGF-2 binds via a specific motif (Phe95, Ser100, Asn102, Arg107, Arg109) [38], which enhances its activity in endothelial proliferation and angiogenesis [39,40]. Mutations disrupting this interaction impair fibrin’s ability to potentiate FGF-2 signaling, highlighting the functional importance of these molecular interfaces. Fibrin(ogen) not only binds growth factors but also protects them from proteolytic degradation [41,42], extending their extracellular half-life [8]. Fibrin matrices establish spatial growth factor gradients that direct cell migration and matrix remodeling [43]. They also promote receptor co-localization, as seen with integrin αvβ3 and FGFR1 [44], which synergistically amplify intracellular signaling. Similar principles apply to VEGF, whose activity is enhanced through ECM interactions [45]. Fibrinogen and fibrin interact with growth factors to modulate their availability, localization, and activity within the extracellular environment. These interactions regulate key cellular responses, including cell adhesion, migration, proliferation, and angiogenesis (Figure 1).

## 5. Functional Implications in Tissue Repair and Angiogenesis

Fibrin(ogen)-bound growth factors act as reservoirs and amplifiers of signaling, supporting angiogenesis, fibroblast function, and tissue repair. Experimental models—including placental explants, chicken chorioallantoic membranes, and murine Matrigel implants—demonstrate that fibrinogen significantly enhances angiogenesis driven by fibrin-bound FGF-2 and IL-1β. Meanwhile, mutants unable to bind fibrinogen show reduced vascularization [46,47].

Growth factors coordinate tissue repair by regulating cell proliferation, migration, and differentiation. PDGF recruits and stimulates fibroblasts and smooth muscle cells for ECM deposition, while EGF promotes keratinocyte proliferation and wound closure [5,48,49]. VEGF and FGFs, particularly FGF-2, drive angiogenesis by promoting endothelial proliferation, migration, tube formation, and matrix remodeling. TGF-β modulates ECM deposition and protease activity, balancing matrix synthesis and degradation for proper tissue architecture [50]. The interplay and timing of these factors ensure coordinated healing, integrating angiogenesis, fibroblast recruitment, and epithelial closure. Dysregulation can lead to chronic wounds, impaired healing, or fibrosis, underscoring the clinical relevance of understanding these mechanisms.

## 6. Fibrinogen in ECM as a Regulator of Growth Factor Activity

Many biological activities require specific interactions with ECM proteins, including development, morphogenesis, and growth. The ECM is composed of several different structures and molecules; the most common are fibronectin, fibrinogen, laminin, collagen, vitronectin, and thrombospondin. Although these proteins differ in primary structure, they retain similar functional motifs that contribute to their adhesive properties for both cells and other proteins, as well as to their ability to organize into fibrillar structures [51]. Fibrinogen colocalizes with fibronectin in the ECM [52]. Further analysis of matrix fibrinogen showed that fibrinogen assembles into matrix as an intact molecule, not as monomeric or polymeric fibrin, suggesting that cryptic “fibrin” specific epitopes are exposed upon fibrinogen incorporation into matrix [53,54]. Much like in a healing wound, the deposition of fibrin(ogen) and other adhesive glycoproteins into the ECM serves as a scaffold to support the binding of growth factors and to promote cellular responses such as adhesion, proliferation, and migration during angiogenesis and tumor cell growth. Reversible binding of growth factors within the ECM enables controlled release through matrix remodeling or mechanical forces [55,56]. Integrin-mediated signaling further integrates ECM composition with growth factor receptor activation, exemplified by αvβ3-dependent enhancement of VEGFR-2 signaling and integrin-mediated activation of latent TGF-β [57,58]. This bidirectional regulation creates a dynamic feedback loop linking ECM remodeling, growth factor signaling, and tissue architecture. These ECM-based mechanisms provide the framework through which fibrin(ogen) integrates molecular signaling with tissue-level repair processes [59].

## 7. Fibrin(ogen) in Growth Factor Regulation and Tissue Repair

Fibrinogen and fibrin are central to hemostasis, extracellular matrix dynamics, and tissue regeneration. They interact with growth factors, localizing them, protecting them, and potentiating their signaling, which directly influences angiogenesis, cell proliferation, and wound healing. Fibrin matrices protect growth factors from proteolytic degradation, extend their extracellular half-life, and establish spatial gradients that guide cell migration and matrix remodeling. They also promote receptor co-localization, exemplified by integrin–growth factor receptor crosstalk, thereby amplifying intracellular signaling [44,60,61]. VEGF activity is similarly enhanced through ECM interactions, demonstrating a broader principle of matrix-mediated growth factor regulation. In tissue repair, growth factors coordinate proliferation, migration, differentiation, and survival. PDGF recruits and stimulates fibroblasts for ECM deposition, EGF promotes keratinocyte proliferation and re-epithelialization, and VEGF and FGFs drive angiogenesis and matrix remodeling. TGF-β balances ECM synthesis and degradation, supporting proper tissue architecture and scar formation. Temporal coordination of these factors ensures effective healing.

Therapeutically, fibrin(ogen) is leveraged as a hemostatic agent, regenerative scaffold, and drug delivery platform. Fibrinogen concentrates and fibrin sealants restore coagulation and promote wound healing [62,63]. These same properties have been harnessed in fibrin-based hydrogels and regenerative scaffolds that provide structural support while simultaneously regulating growth factor availability and cellular signaling. Modulating fibrin architecture, degradation kinetics, or growth factor binding capacity represents a promising strategy to enhance tissue regeneration while limiting pathological remodeling [64,65]. Together, these applications position fibrin(ogen) as both a physiological regulator of growth factor signaling and a versatile therapeutic material linking hemostasis, regeneration, and disease modulation.

## 8. Fibrin Biomechanics, Scaffolds, and Growth Factor Availability

The biomechanical properties of fibrin are central to its biological function and have been extensively studied in the context of clot stability, wound healing, and cell–matrix interactions. Parameters such as fiber diameter, network density, stiffness, and viscoelasticity are influenced by fibrinogen concentration, thrombin activity, factor XIII-mediated crosslinking, and mechanical forces within the tissue environment [66]. These properties vary depending on fibrinogen concentration, polymerization conditions, crosslinking, and local mechanical forces, leading to considerable heterogeneity in fibrin structure across tissues and contexts. These structural and mechanical features are known to regulate cell adhesion, migration, and mechano-transduction through integrin-mediated interactions, highlighting fibrin as a dynamic and responsive extracellular matrix rather than a passive scaffold.

Fibrin-based scaffolds have been widely used in experimental and translational settings, where matrix mechanics and architecture can be deliberately tuned. Adjustments in fibrin concentration, polymerization conditions, and crosslinking are commonly employed to modulate scaffold stiffness, porosity, and degradation behavior, primarily to influence cellular responses [67]. Several studies have shown that fibrin structure affects molecular transport and proteolytic accessibility within the matrix, suggesting that biomechanical properties may indirectly shape the local biochemical environment [68,69]. Fibrin, either alone or in combination with other biomaterials, has been widely employed as a biological scaffold to support stem or primary cells for the regeneration of diverse tissues, including adipose, bone, cardiac, cartilage, liver, neural, ocular, skin, tendon, and ligament tissues [70,71].

Although direct experimental evidence remains limited, it is plausible that fibrin biomechanics and scaffold architecture also influence the availability of fibrin-associated growth factors by altering binding site exposure, diffusional constraints, and release during fibrinolysis.

Several preclinical studies have demonstrated the feasibility of incorporating growth factors into fibrin-based scaffolds for tissue repair applications [72,73]. For example, fibrin scaffolds combined with concentrated growth factors and stromal vascular fractions have been shown to improve structural and functional outcomes in experimental models of musculoskeletal injury, such as chronic rotator cuff tears [74]. While these studies primarily evaluate regenerative efficacy rather than underlying mechanisms, they provide proof of principle that fibrin matrices can serve as clinically relevant carriers for bioactive factors. Clarifying how mechanical tuning of fibrin scaffolds affects growth factor retention and release represents an important area for future investigation.

## 9. Pathological Roles of Fibrin(ogen)

Dysregulation of fibrinogen expression, fibrin deposition, or fibrinolysis transforms a normally self-limiting repair system into a driver of pathology. Persistent fibrin matrices alter growth factor availability, sustain inflammatory signaling, and disrupt tissue remodeling across inflammatory, fibrotic, thrombotic, and neoplastic diseases. In chronic inflammatory diseases, prolonged fibrin persistence plays a central pathogenic role. Instead of being efficiently cleared after its reparative function, fibrin accumulates within tissues and continuously activates immune cells. Fibrinogen interacts with integrin receptors such as αMβ2 on leukocytes, stimulating the release of pro-inflammatory cytokines, chemokines, and reactive oxygen species [75]. This sustained immune activation disrupts normal tissue repair and promotes progressive damage. In diseases like rheumatoid arthritis, fibrin deposits within the synovium amplify joint inflammation and destruction [76]. In multiple sclerosis, fibrin leakage across the damaged blood–brain barrier activates microglia, contributing to neuroinflammation and neuronal injury [77,78]. Similarly, in chronic lung diseases, fibrin-rich exudates impair gas exchange and drive fibrotic remodeling [79].

Fibrotic disorders represent a pathological extension of fibrin-driven repair mechanisms. Persistent fibrin matrices provide a platform for excessive activation of growth factors such as TGF-β, a master regulator of fibrosis. This environment promotes fibroblast differentiation into myofibroblasts, cells responsible for excessive collagen and extracellular matrix production. Over time, the normal tissue architecture is replaced with stiff, nonfunctional scar tissue. In organs such as the liver, kidneys, and lungs, this process leads to irreversible loss of function. Rather than resolving injury, fibrin-mediated signaling locks tissues into a chronic wound-healing state, highlighting how a normally beneficial process becomes maladaptive in disease.

**Figure 2:**
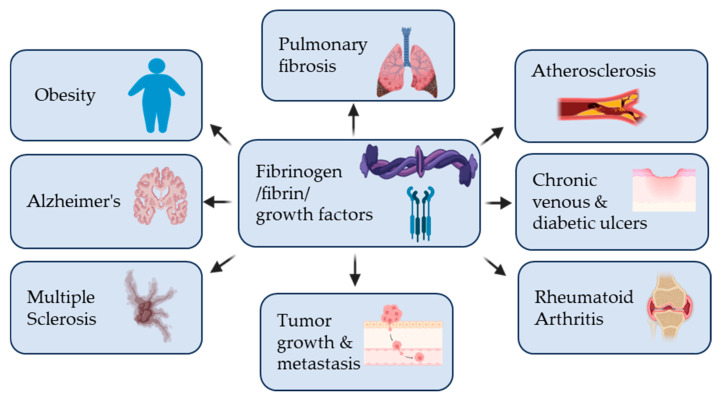
Fibrinogen/Fibrin and growth factors in human diseases. Fibrinogen and its insoluble polymerized counterpart fibrin, along with degradation products of fibrin, are involved in the pathogenesis of numerous diseases. Aberrant fibrin(ogen) deposition, persistence, or degradation contributes to numerous disease progressions by altering coagulation, inflammation, and cell–matrix interactions across multiple tissues. Representative disease illustrations are from BioRender.com (https://app.biorender.com/illustrations/69431ba20505fa63df077a7b (accessed on 19 February 2026)).

Abnormal fibrin regulation is also central to thrombotic diseases, where excessive clot formation or insufficient fibrinolysis leads to vascular occlusion. Fibrin-rich clots are not biologically inert; they actively interact with endothelial cells, platelets, and immune cells. Within thrombi, fibrin binds growth factors and inflammatory mediators that exacerbate vascular injury and delay endothelial repair. In ischemic stroke and myocardial infarction, this contributes to secondary tissue damage beyond the initial loss of blood flow. In atherosclerosis, fibrinogen infiltrates damaged vessel walls, stimulating smooth muscle cell proliferation and promoting plaque growth [80]. These effects increase plaque fragility, raising the risk of rupture and acute cardiovascular events. Fibrin(ogen) plays a pivotal role in the development and progression of several human disorders, extending beyond coagulation to influence inflammatory responses, tissue remodeling, and disease pathogenesis across multiple organ systems. (Figure 2). Various inflammatory and prothrombotic diseases share common alterations in fibrinogen expression, fibrin structure, and fibrinolytic balance. Conditions such as sickle cell disease, infection and sepsis, obesity, ischemic stroke, Alzheimer’s disease, and chronic non-healing wounds are frequently associated with elevated circulating fibrinogen and enhanced fibrin deposition [81,82,83,84,85,86]. In these settings, fibrinogen contributes to increased clot density, resistance to fibrinolysis, and persistent fibrin accumulation, promoting vascular occlusion, impaired tissue perfusion, and sustained inflammation. Beyond its role in coagulation, fibrinogen interacts with endothelial cells, immune cells, and platelets, amplifying inflammatory signaling and tissue damage. Collectively, these observations highlight fibrinogen as a central mediator linking chronic inflammation to prothrombotic pathology across diverse disease contexts.

Cancer progression is profoundly influenced by fibrinogen and fibrin through their effects on growth factor regulation and the tumor microenvironment. For example, fibrinogen produced by cancer cells enhances the proliferative effects of FGF-2 [87]. Tumor cells can actively shape their microenvironment by synthesizing and secreting fibrinogen locally to support primary tumor growth. Many tumors hijack coagulation pathways to induce localized fibrin deposition, effectively creating a supportive scaffold for tumor expansion. Fibrin binds angiogenic growth factors such as VEGF and FGF, intensifying blood vessel formation within tumors and supporting their high metabolic demands. This fibrin-rich matrix also promotes tumor cell migration and invasion by providing adhesive surfaces and activating integrin-mediated signaling pathways [88]. In addition, fibrinogen promoted trans-endothelial migration exclusively in malignant breast cancer cells. Meanwhile, nonmalignant breast epithelial cells were unaffected, highlighting intrinsic differences in fibrin(ogen) interactions between cancerous and normal cells [89]. Inflammatory signals amplified by fibrin(ogen) further enhance tumor growth, linking coagulation, inflammation, and cancer biology. Overall, Fibrinogen supports locally invasive tumor growth by forming a persistent provisional matrix within the tumor microenvironment, while vascular leakiness and local coagulation lead to fibrin deposition at invasive fronts, which provides a mechanically permissive substrate for integrin-mediated tumor cell migration. In addition, fibrin sequesters angiogenic and stromal growth factors at sites of matrix remodeling. Protease-mediated release of these factors reinforces tumor cell motility, angiogenesis, and stromal activation, while sustained fibrin deposition promotes chronic inflammation and immune modulation.

Fibrinogen plays a particularly important role in cancer metastasis [90,91,92]. Earlier studies have shown that fibrinogen and thrombin may work cooperatively to facilitate metastatic disease but have no apparent effect on tumor cell proliferation [92]. This further suggests that circulating plasma fibrinogen plays a critical role in tumor dissemination and metastasis. Meanwhile, primary tumors can rely on fibrinogen produced within the tumor itself, independent of plasma levels. Moreover, the interaction between FGF-2 and fibrinogen may contribute to both the expansion of primary tumors and the spread of cancer to distant sites (metastasis). Fibrinogen-mediated adhesion to endothelial cells supports extravasation into secondary organs, where fibrin matrices again help establish a permissive niche for metastatic growth. Recently, a study revealed that fibrin(ogen) plays a multifaceted role in metastasis, promoting tumor cells’ entry into the vasculature from a primary tumor while impeding the early survival of circulating tumor cells once in the vasculature [93]. Clinically, elevated fibrinogen levels correlate with advanced disease stage, increased metastatic burden, and reduced survival in multiple cancers, underscoring its value as both a biomarker and a therapeutic target.

In impaired wound healing, such as diabetic ulcers or chronic pressure wounds, fibrin becomes a pathological barrier rather than a regenerative scaffold. Excessive or persistent fibrin disrupts normal growth factor gradients and prevents effective cell migration necessary for re-epithelialization and neovascularization. The resulting hypoxic and inflammatory environment further suppresses proper healing, creating a vicious cycle of tissue breakdown. Across cancer, inflammatory disorders, thrombosis, fibrosis, and chronic wounds, fibrin(ogen) emerges as a dynamic regulator of growth factor activity whose dysregulation drives disease. Targeting fibrin-related pathways, therefore, holds promise for therapeutic intervention across a broad spectrum of pathophysiological conditions.

## 10. Conclusions, Future Directions, and Unanswered Questions

The primary aim of this review is to introduce and integrate the emerging concept of fibrinogen and fibrin as regulators of growth factor activity, with emphasis on biological and pathological relevance. To our knowledge, this manuscript represents the first review specifically focused on fibrinogen and fibrin as regulators of growth factor activity. Recent advances in vascular biology have reshaped our understanding and perception of fibrinogen and its polymerized form, fibrin. Beyond their traditional role as structural end-products of the coagulation cascade, these proteins are now widely accepted as regulators of growth factor activity. Emerging evidence suggests that fibrin(ogen) functions as a high-affinity binding matrix for key growth factors, including VEGF, FGF-2, and TGF-β, to localize, stabilize, and gradually control their release and bioavailability. This aspect of fibrin(ogen) in sequestering and delivering growth factors has important implications for tissue repair during wound healing and angiogenesis. However, persistent fibrin deposition and sustained growth factor activation have been linked to various pathological processes, including fibrosis, tumor progression, chronic inflammatory states, and many more. Therefore, fibrin(ogen) functions not merely as a structural scaffold but as a dynamic regulator of growth factor activity through cellular signaling pathways that drive disease progression. Elucidating the molecular mechanisms underlying fibrin(ogen)–growth factor interactions reveals promising therapeutic avenues. Strategic targeting of this interactome could enable precise modulation of regenerative processes while simultaneously curbing maladaptive signaling across a broad spectrum of pathological conditions.

Research on fibrinogen and fibrin continues to expand rapidly, driven by advances in molecular biology, biomaterials engineering, and regenerative medicine. Despite significant progress, important gaps remain that warrant further investigation.

A key area for future research is the regulation of growth factor interactions with fibrin matrices. Although fibrin is known to bind and protect growth factors such as FGF, VEGF, PDGF, and TGF-β, the mechanisms governing their spatial and temporal availability in vivo remain incompletely defined. Greater insight into how matrix composition, crosslinking density, and fibrin degradation influence growth factor release could inform the rational design of more precise and effective therapeutic strategies.

Another emerging direction is the development of personalized and tissue-specific approaches. Most current fibrin-based systems rely on standardized formulations, yet variability in fibrinogen levels, growth factor expression, and tissue microenvironments may substantially affect biological outcomes. Tailoring fibrin-based materials to patient-specific or tissue-specific contexts may enhance efficacy in applications such as wound repair, angiogenesis, and tissue regeneration.

Recombinant fibrinogen and synthetic fibrin-mimetic materials also present promising opportunities but pose technical challenges. Achieving consistent growth factor binding, predictable degradation behavior, and low immunogenicity remains a key objective. Future efforts will likely focus on integrating these materials with engineered hydrogels, composite scaffolds, or stimuli-responsive systems to enable controlled delivery of growth factors and cells.

At the molecular level, several fundamental questions remain unresolved, including how fibrinogen-mediated signaling interfaces with integrins and growth factor receptors, and how these interactions vary across cell types and disease states. Elucidating these mechanisms may enable the development of targeted modulators that promote tissue repair while limiting adverse outcomes such as fibrosis, thrombosis, or tumor progression.

Finally, successful clinical translation will require systematic evaluation of safety, reproducibility, and long-term outcomes. Optimizing growth factor stability, matrix degradation kinetics, and integration with host tissue will be essential for fully realizing the therapeutic potential of fibrin(ogen)-based strategies. Addressing these challenges will help bridge fundamental mechanistic insights with innovative biomaterial design and translational regenerative applications.

## Figures and Tables

**Table 1:** Growth Factors in Relation to Fibrinogen and Fibrin. The table summarizes reported binding interactions (“Yes” or “No”) for various growth factors as described in the cited references. It does not reflect binding affinity, isoform specificity, context dependence, or mechanistic details. Multiple ligands listed in a single row indicate that the same binding behavior was reported for all listed isoforms. “No/Yes” indicates conflicting or context-dependent findings among studies. Reference numbers correspond to the literature sources cited.

Growth Factors	Binding	Reference
FGF-1	No	[5,8]
FGF-2	Yes	[4,5]
FGF-4, 6, 8, 9,10,18	No	[5]
FGF-5, 7	Yes	[5]
VEGF	Yes	[5,6]
VEGF-A165, -A121, -C	No	[5]
PDGF-AB, -BB, -DD	Yes	[5]
PDGF-AA, -CC	No	[5]
PlGF-2 and PlGF-3	Yes	[5]
PlGF-1	No	[5]
TGF-β1, TGF-β2	Yes	[5]
TGF-β3	No	[5]
IGF	No	[5]
EGF	No/Yes	[5,9]
NGF	No	[5]
IL-1β	Yes	[7]
IL-1α	No	[7]

## Data Availability

No new data was created or analysed during this study. Data sharing is not applicable to this study.

## Abbreviations

The following abbreviations are used in this manuscript:

FGF	Fibroblast growth factor
VEGF	Vascular endothelial growth factor
PDGF	Platelet-derived growth factor
TGF	Transforming growth factor-beta
ECM	Extracellular matrix
PlGF	Placenta growth factor
NGF	Nerve growth factor
EGF	Epidermal growth factor
IGF	Insulin-like growth factor
IL-1	Interleukin-1
